# Bypassing shortages of personal protective equipment in low-income settings using local production and open source tools

**DOI:** 10.1371/journal.pbio.3001658

**Published:** 2022-05-20

**Authors:** Royhaan Olamide Folarin, Mahmoud Bukar Maina, Abisola Kaosara Akinbo, Tamramat Iyabo Runsewe-Abiodun, Omobola Abioye Ogundahunsi, Ahmed Adebowale Adedeji, Andre Maia Chagas

**Affiliations:** 1 Department of Anatomy, Olabisi Onabanjo University, Sagamu (Ogun State), Nigeria; 2 TReND in Africa, Brighton, United Kingdom; 3 Sussex Neuroscience, School of Life Sciences, University of Sussex, Brighton, United Kingdom; 4 Biomedical Science Research and Training Centre, Yobe State University, Nigeria; 5 Department of Paediatrics, Faculty of Clinical Sciences, Olabisi Onabanjo University, Sagamu (Ogun State), Nigeria; 6 Department of Chemical Pathology, Faculty of Basic Medical Sciences, Olabisi Onabanjo Univeristy, Sagamu (Ogun State), Nigeria; 7 Department of Pharmacology, Faculty of Basic Medical Sciences, Olabisi Onabanjo University, Sagamu (Ogun State), Nigeria

## Abstract

Free and open-source hardware, 3D printing and the use of locally sourced materials are valuable tools for local problem solving. This Community Page describes how PPE supply chain problems could be bypassed using open science in a Nigerian community.

## Teaser

Free and open-source hardware, 3D printing, and the use of locally sourced materials can be valuable tools for local problem solving, as proven by the production of more than 400 reusable face shields and masks in a Nigerian community to bypass PPE shortages during the COVID-19 pandemic.

## Body text

As the infection rates of Coronavirus Disease 2019 (COVID-19) rose globally, personal protective equipment (PPE) shortages led to an increased risk of infection and decreased effectiveness in health response systems [1]. Prices for surgical masks, respirators, and surgical gowns hiked, and some countries blocked PPE exports to guarantee national access [1], while others reportedly intercepted international shipments and delivered them to the highest bidder [2]. To help tackle this issue, alternative means of achieving PPE production and distribution were required, and the utility of free and open-source hardware (FOSH) and 3D printing quickly became evident, particularly for low-income settings where the resources to compete globally to import PPE are usually limited. Following other global initiatives [3] and demonstrating the usefulness of this approach, FOSH designs were successfully used to locally produce face shields and masks using 3D printing and relatively accessible and inexpensive materials for distribution to communities around a Nigerian University during the second trimester of 2020.

**Fig 1:**
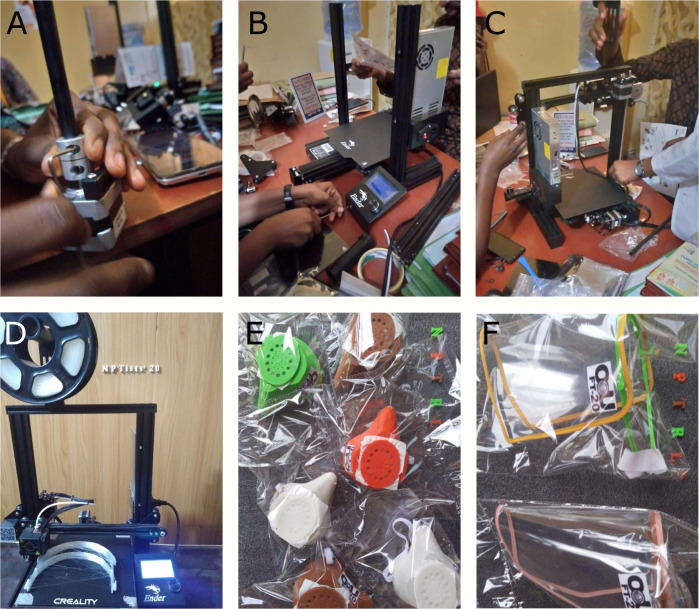
3D printer assembly and produced PPE. (A-D) Different snapshots while building the 3D printer from the very beginning (A) to the complete working printer (D). (E) Complete and packaged face masks. (F) Complete and packaged face shields.

The fourth industrial revolution [4] has enabled digital designs to be seamlessly shared over the internet and replicated everywhere, and 3D printing has become an affordable and easily accessible tool for the fabrication of alternatives to rather expensive and inaccessible hardware. This production method is resistant to traditional supply chain problems, as the raw materials needed for 3D printing can be used for many different applications and stored in bulk over extended periods of time. Using this method, we were able to manufacture over 400 face shields and masks (Fig 1) using design files downloaded from different repositories, Thingiverse, Youmagine, and Printables (face shield designs by 3DVerkstan and Ric Kolibar; masks designs by Drew Dupont and Prusa). The files were modified for sizing and branding as desired using the web-based Tinkercad program and STL files were sliced into g-code using Slic3r and uploaded for printing. One 3D printer operator and one assembler produced on average 1 face shield in 1 hour 30 minutes, costing 1,200 Naira (2.92 USD), and 1 mask in 3 hours 30 minutes, costing 2,000 Naira (4.86 USD). Labour constituted 23% of the cost for the mask and 31% of the cost for the face shield (details of production can be found in [5]). At the time of the project, commercially available face shields and reusable masks cost at least 5,000 Naira (12.16 USD) and 10,000 Naira (24.32 USD), respectively.

Our 3D printer was not built for massive serial manufacturing (as seen in the time taken to print each item); however, several 3D printers running in parallel can be used to reduce relative production time, as shown by the company Prusa Research, which produced, CE certified, and shipped 200,000 face shields using their own print farm [6]. With only one printer, we worked as a bridge, supplying as many items of PPE as we could until traditional manufacturers could fulfil the demand. After initial use, oral feedback from testers commended innovativeness, usefulness, and aesthetics of the produced PPE. At the initial production and distribution stages, this feedback helped to improve the skin-friendliness of the shields, by adjusting smoothness, band size, and general finishing. The PPE was useful to hospital health workers, paramedics, first responders (police officers and firefighters), dentists, laboratory workers, veterinarians, custodial staff dealing with spills and contaminated waste, grocery store staff, as well as those providing essential services or in contact with many people during the pandemic.

Producing PPE locally came with some challenges. Overall, the cost of procuring and shipping the Ender-3 pro 3D Printer (Shenzhen Creality 3D Technology, Shenzhen, China) at the time of the project was 190,000 Naira (457.17 USD). 3D printing filament made of polylactic acid (PLA) or polyethylene terephthalate glycol (PETG) was also procured from China at a cost of 150,000 Naira (360.79 USD) per 50 kg of PLA filament (Box 1). We found that issues with 3D printing could slow down production and familiarity with them and the general machine operation were paramount for consistency. Fortunately, as part of the Alumni network of Trend in Africa, we were in contact with kind and willing experts from across the globe who were ready and able to offer support and ideas when necessary through online communication. Power cuts from the main grid occurred (something common in Nigeria), leading to many prints stopping in the middle of production. With acquired expertise, we were able to manoeuvre production to continue building from exactly where they had stopped and avoid wasting an incomplete print.

**Box 1. Sourcing materials for local production in low-income settings**

- Sourcing materials for producing PPE or any other type of designs is not trivial in low-income settings. The following are insights gained from our own project that might be helpful to others.
- Do a deep dive into your design documentation. Understanding what all of the components are doing is key to knowing what and how parts can be substituted. When in doubt, and if your project is based on open source designs, asking the original developer is a great way to start a conversation and possibly a long-term collaboration!
- Find local suppliers that might stock the parts you need and do not hesitate to send them your full list of materials as, given their knowledge, they will likely have an easier time locating things and the cost for this service could be worth the saving in time.
- Shopping online is a good way to acquire supplies if you can afford to wait for shipping time and potentially needing to deal with customs paperwork/bureaucracy.
- For mechanical parts that are too heavy/too bulky to ship or that have high costs, finding local artisans (metal workshops, carpenters, etc.) who can manufacture them is a good way to reduce costs.
- It is ok to reach out to experts whenever “sticky problems” appear. Sending a short, polite, and to the point message asking for help/support is a good way to start a conversation. From our experience, people are really open to sharing their knowledge and expertise.

3D printing and FOSH have great potential to fill technological gaps, but more awareness is required in the global South, particularly in Africa [3]. In African universities and research institutions, innovative alternatives to expensive equipment are needed as African research is largely dependent on foreign aid for advancement [7]. This financial dependence makes it impossible to sustainably build and maintain state-of-the-art facilities, as funds do not come at regular intervals [8]. Indeed, foreign aid has been blamed for the developed culture of dependency in Africa, which has consequently fostered paternalism rather than partnership with the global North [7–9]. African scientists therefore have to think innovatively if they are to bring local research/medical infrastructure to the same levels experienced in the global North. Investing in FOSH for local knowledge and capacity building would be one way to innovate, as leveraging open source technologies allows for acquisition of locally sourced components [3], local production of tools, bypassing long waits and bureaucracy with customs, local repair and calibration, reduced costs, and easier customization of existing tools [10]. African governments are thus encouraged to provide the necessary support to increase access to FOSH for African scientists to solidify the foundation needed to gradually wean the continent off the age-long dependence on foreign aid. Consistency in electricity supply and training support for further skill acquisition would be useful additional steps.

As the world tackles COVID-19, the Nigerian project highlighted in this Community Page demonstrates the community-oriented efforts of academics in the local fabrication of 3D-printed face shields and masks. The success of the project further substantiates the fact that even complex pieces of equipment are only an assembly of different simple components and that open designs show how these components go together. Furthermore, the low cost of the required consumables for such projects, such as printing filament, allows innovators to combine funds to start designing substantial solutions for societal problems that were normally only addressed with funds from government/research institutions. We hope this project will inspire other communities in low- and middle-income countries to follow a similar approach for the opportunities present within their respective domains, just as it was deployed successfully in this case for responding promptly to a health emergency. By leveraging FOSH projects and initiatives, further breakthroughs are expected in the fabrication of scientific equipment and test kits needed for medical diagnostics, building on what groups have already achieved in high-income countries.

## Abbreviations:

COVID-19: Coronavirus Disease 2019
FOSH: free and open-source hardware
PETG: polyethylene terephthalate glycol
PLA: polylactic acid
PPE: personal protective equipment
